# MIGC: Pseudotime differentially expressed genes detection with mutual information

**DOI:** 10.1002/qub2.70057

**Published:** 2026-10-19

**Authors:** Jiachen Li, Ye Yuan

**Affiliations:** ^1^ State Key Laboratory of Biopharmaceutical Preparation and Delivery Institute of Process Engineering Chinese Academy of Sciences Beijing China

**Keywords:** DEG detection, pseudotime analysis, scRNA‐seq analysis

## Abstract

Trajectory analysis of single‐cell RNA sequencing (scRNA‐seq) data provides insights into cell differentiation and the underlying mechanisms at the gene level. Although various pseudotime inference methods have been proposed, the detection of pseudotime differentially expressed genes (DEGs) mainly relies on generalized additive model fitting followed by statistical hypothesis testing. However, considering that pseudotime inference is a prerequisite for pseudotime DEG detection, it would be beneficial to unify them into an integrated framework. Inspired by the concepts of information theory, we propose a trajectory analysis strategy called MIGC by capturing the mutual information between genes and cells, which can not only accurately infer cell pseudotime but also identify pseudotime DEGs. Experiments on multiple scRNA‐seq datasets demonstrate that MIGC offers an effective trajectory analysis pipeline that outperforms baseline approaches in detecting pseudotime DEGs.

## 1 INTRODUCTION

Single‐cell RNA sequencing (scRNA‐seq) [1, 2] has become a revolutionary tool in the field of molecular biology, offering insights into cellular heterogeneity and dynamics at single‐cell resolution. The analysis of scRNA‐seq data can be performed in both the cell domain and the gene domain. On the one hand, cell state analysis is a fundamental task for single‐cell analysis. For instance, for datasets describing differentiation or developmental processes, pseudotime [3] inference is always performed. On the other hand, selecting genes that might have important functions is also critical, known as highly variable gene (HVG) and differentially expressed gene (DEG) selection in current protocols.

Pseudotime inference aims to infer the ordering of cells along a lineage from scRNA‐seq data. Multiple computational methods have been proposed for this task, including PAGA [4], Monocle [5], Slingshot [6], and Palantir [7]. Many of them employ graph models to construct a Markov chain for trajectory generation, with pseudotime determined according to the initial cluster or root node defined by the user [8]. In recent years, RNA velocity [8, 9, 10] methods have been introduced to infer pseudotime without requiring the annotation of initial cell states by modeling the dynamics between unspliced mRNA and spliced mRNA. Nevertheless, because of the high noise and sparsity in scRNA‐seq data, such modeling may not be able to accurately fit the dynamics of many genes [11]. Considering that only a subset of genes in scRNA‐seq data could play significant roles in differentiation, once pseudotime is derived for a scRNA‐seq dataset, detecting the genes whose expression changes most substantially with pseudotime constitutes a core downstream task [12] and commonly conducted in previous pipelines [5, 6, 7, 13, 14, 15]. The genes with pseudotime‐specific expression patterns are regarded as pseudotime DEGs. Pseudotime DEG recognition can not only help verify computational methods of dynamics modeling but also provide biological findings in cellular developmental processes.

For pseudotime DEG detection tasks where a continuous representation of cell state is provided, the generalized additive model (GAM) is widely adopted in previous approaches [12, 13, 15]. In existing approaches, gene expression is first modeled as a combination of smoothed functions of time using GAM. DEGs are then identified by testing whether the combined function changes over pseudotime. However, this two‐step process may overlook the interdependencies between gene expression and pseudotime. As a result, the top‐ranked DEGs selected by these methods may not be fully interpretable within the context of the cellular processes represented in the corresponding scRNA‐seq datasets. In addition, given that pseudotime inference is fundamental for identifying pseudotime DEGs, integrating gene expression modeling and pseudotime analysis into a single framework would be beneficial. In this study, we propose a novel gene selection method that evaluates the interrelationship between gene expression and cell states.

Based on our analysis of the relations between genes and cells, we propose the idea that genes with high mutual information (MI) scores with pseudotime are supposed to be DEGs. In recent years, MI has been employed in single‐cell analysis for multiple tasks, including multiomic integration [16], cell cycle‐related gene detection [17], dimensionality reduction [18], and gene regulation analysis [19]. We present a pseudotime analysis framework termed MIGC (maximizing MI between genes and cells), which simultaneously achieves cell pseudotime inference and pseudotime DEG identification. Experimental results on various scRNA‐seq datasets suggest that pseudotime enables reliable inference using MIGC and genes that play significant roles in differentiation can also be detected. Compared with the previous pseudotime analysis pipeline for scRNA‐seq, MIGC can find genes that better align with the biological process and provide more interpretable results.

## 2 RESULTS

### 2.1 Overview of the MIGC framework

Because of the noisy and high‐dimensional nature of scRNA‐seq data, selecting HVGs is crucial for downstream analysis. Considering that entropy quantifies intrinsic data uncertainty, we systematically compared the expression entropy and variance of individual genes across four independent scRNA‐seq datasets and identified a strong positive correlation between the two metrics (Spearman’s *ρ* > 0.99; Supporting Information S1: Figure S1). This result indicates that entropy can effectively serve as a proxy for HVG identification. However, high variance and entropy do not necessarily reflect biological informativeness, as they can stem from noise. For example, we simulated two genes with identical marginal distributions along pseudotime (Figure 1A). Despite comparable entropy and variance, only one gene exhibits clear pseudotime dependency, whereas the other is dominated by noise. This distinction is reflected in their joint distributions, where the informative gene shows lower conditional entropy H(G|T). Accordingly, gene informativeness can be characterized by high H(G) and low H(G|T). MI, MI(G,T)=H(G)−H(G|T), provides a natural measure of this property, with higher MI indicating stronger pseudotime association.

**FIGURE 1:**
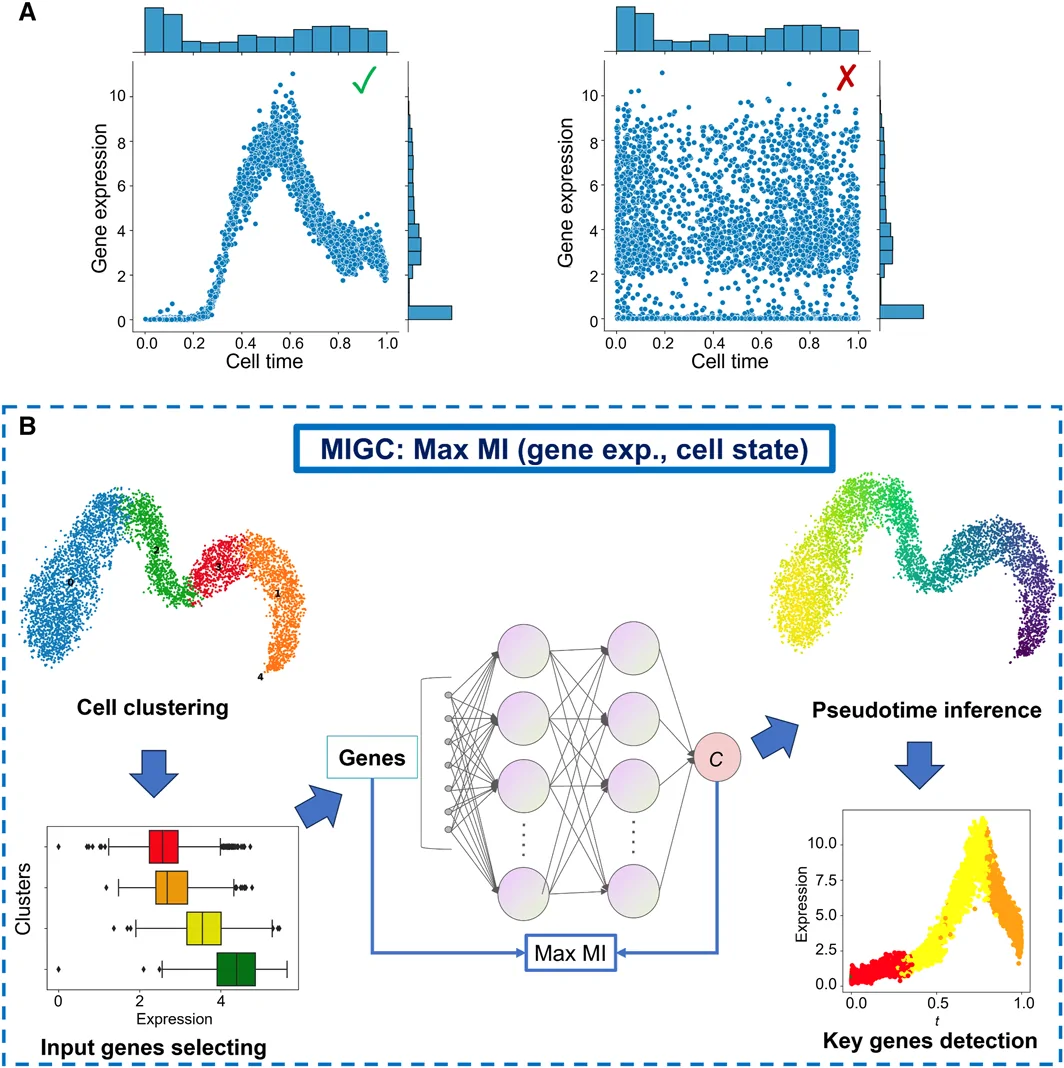
Overview of MIGC. (A) A pair of examples, which share the same variance and entropy, for illustrating the more and less informative genes with respect to time. (B) The pseudotime analysis pipeline with MIGC, which includes both pseudotime inference and pseudotime differentially expressed gene detection. Taking gene expressions as input, the neural network outputs a one‐dimensional cell embedding C, which could be regarded as pseudotime. The loss function is designed to maximize the mutual information (MI) between the generated cell embedding C and the expression of each gene.

The insight about maximizing the MI between gene expression and cell state representation could be generalized to various tasks. On the one hand, given a fixed cell state representation, for example, clusters or pseudotime, the task‐specific DEGs could be selected by $\underset{\text{gene}}{\max\;}\text{MI}\left(\text{gene}\;\text{expression},\text{cell}\;\text{state}\right)$. On the other hand, using a fixed set of genes, the cell state representation could be learned by $\underset{\text{cell}\;\text{state}}{\max\;}\text{MI}\left(\text{gene}\;\text{expression},\text{cell}\text{state}\right)$. This enables pseudotime inference on developmental trajectories. To improve the internal consistency and self‐consistency of the pseudotime analysis strategy, the MIGC pipeline integrates both applications for pseudotime inference and DEG selection (Figure 1B), with detailed steps provided below.

First, we preprocessed the raw count data and conducted clustering to group the cells and manually determined the root cluster, which is also a step required in most previous pseudotime inference methods [4, 5, 6, 7]. Next, we calculated the MI between each gene’s expression and cell clusters, identifying the genes that have high MI with clusters. Considering pseudotime as a continuous cell state representation and cell clusters as discrete ones, this step helped filter out those noisy genes with weak signals for cell state identification and also enhanced the speed of downstream modeling. The selected genes were then fed into a neural network, which generates a one‐dimensional cell embedding as output. The loss function is defined as the negative mean MI between the input gene expressions and the output cell embedding. After training, the optimized cell representation was obtained, maximizing MI with the input gene expressions. This resulting cell representation can be interpreted as an MI‐based pseudotime (MIt). Finally, genes associated with high MI between their expression and the learned MIt are regarded as pseudotime DEGs.

To incorporate MI into the loss function for model training, a fast, accurate, and differentiable estimator of MI for continuous variables is required. Direct computation of MI from its mathematical definition or using most existing estimators is not suitable, as they do not support gradient‐based optimization. Accordingly, we adopted the relative squared loss MI (RSMI) [20], which enables efficient and gradient‐preserving estimation. The estimated RSMI (ERSMI) is employed throughout our work as an efficient estimator for MI computation between continuous variables. More details about the MIGC framework, RSMI, and its estimation are provided in the Methods.

### 2.2 MIGC outperforms baseline approaches for pseudotime DEG detection on the mouse brain dataset

First, we applied MIGC to an embryonic E18 mouse brain scRNA‐seq dataset from 10× Multiome for both pseudotime inference and pseudotime DEG detection. As described in a previous study [21], radial glial cells are capable of division, producing intermediate progenitor cells that serve as neural stem cells and give rise to various mature excitatory neurons in cortical layers. The development of cortical layers follows an inside‐out pattern during neuronal migration, wherein younger cells ascend to upper layers, whereas older cells remain in deeper layers (Figure 2A). As shown in Figure 2B, the inferred MIt can align well with the ground‐truth developmental process.

**FIGURE 2:**
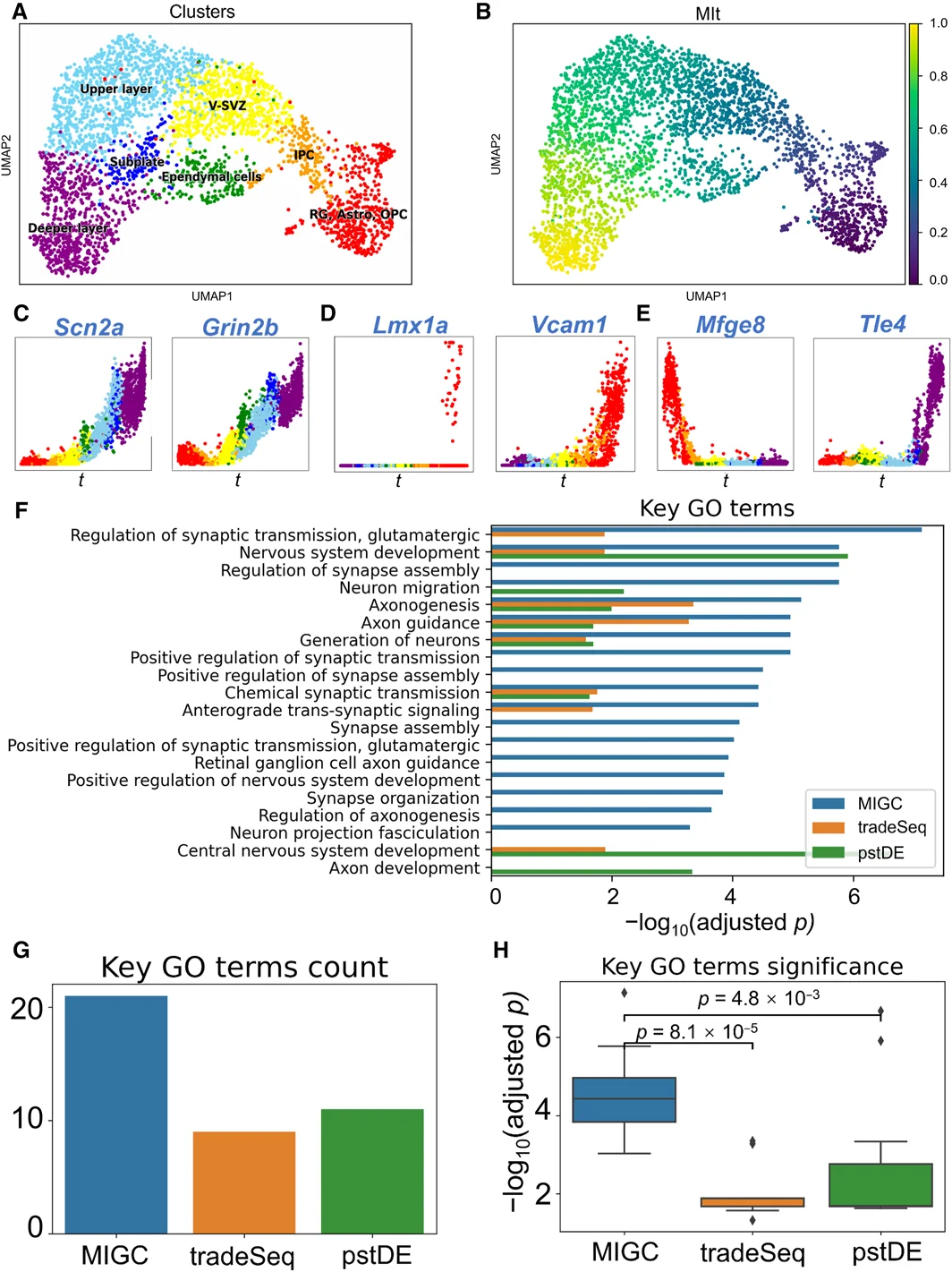
Results of MIGC on the 10× embryonic mouse brain dataset. (A) The cell type annotation. (B) The inferred pseudotime representation MIt. (C) The expression of *Scn2a* and *Grin2b* along MIt, which are the top‐ranked pseudotime DEGs selected by MIGC. (D) The expression of *Lmx1a* and *Vcam1* along the Slingshot pseudotime, which are the top‐ranked DEGs selected by pstDE. (E) The expression of *Mfge8* and *Tle4* along the Slingshot pseudotime, which are the top‐ranked DEGs selected by tradeSeq. (F) The comparison of key GO terms, which refer to those GO terms related to the nervous system, enriched in the top‐100‐ranked genes detected by MIGC, tradeSeq, and pstDE. (G) The comparison of the number of key GO terms obtained by MIGC, tradeSeq, and pstDE. (H) The comparison of the significance of key GO terms obtained by MIGC, tradeSeq, and pstDE. DEGs, differentially expressed genes; pstDE, pseudotimeDE.

Additionally, the pseudotime DEGs selected by MIGC can be well interpreted based on their function. For instance, *Scn2a* and *Grin2b*, the top‐2 genes with the highest MI with the learned MIt (Figure 2C), have been reported to be important for the nervous system [22, 23, 24]. In contrast, pseudotimeDE [12] (Figure 2D) and tradeSeq [15] (Figure 2E) tend to identify genes with expression patterns restricted to a short time window as top‐ranked pseudotime DEGs. This is because they use a hypothesis‐testing framework that tests whether genes do not change over time, which could give high significance to genes with transient expression peaks. However, such genes may not be ideal for capturing the full differentiation process represented in scRNA‐seq data, as further supported by the gene function analysis below.

To quantitatively compare the genes selected by MIGC and baseline approaches, we performed GO [25, 26] biological process terms enrichment analysis based on the top‐100 genes selected by MIGC, tradeSeq, and pseudotimeDE. The comparison of GO terms related to the nervous system is shown in Figure 2F, where MIGC has advantages in both the number of GO terms (Figure 2G) and the *p*‐value (Figure 2H). The differences are statistically significant under the Mann–Whitney *U* test (*p* = 4.8 × 10^−3^ and *p* = 8.1 × 10^−5^, respectively).

### 2.3 MIGC can get consistent findings on multiple mouse brain datasets

We applied MIGC to another 10× mouse brain dataset to verify its robustness. The dataset is from the hindbrain (pons) of adolescent mice (Figure 3A), which depicts the differentiation trajectory from oligodendrocyte precursor cells (OPCs) to committed oligodendrocyte precursors (COPs), followed by progression to newly formed oligodendrocytes (NFOLs) and ultimately to mature myelin‐forming oligodendrocytes (MFOLs). By maximizing the MI between pseudotime and gene expression, the generated pseudotime MIt can identify the whole differentiation process (Figure 3B). After that, we calculated the MI between the expression of each gene and MIt and found that the top‐5 pseudotime DEGs are *Ank3*, *Clic4*, *Ermn*, *Ddr1*, and *Kndc1*, respectively (Figure 3C), all of which have been reported to be significant for the nervous system [27, 28, 29, 30, 31]. We also applied tradeSeq and pseudotimeDE to identify pseudotime DEGs and performed GO enrichment analysis using the top‐100 DEGs from each method. Focusing on GO terms related to the nervous system, MIGC identifies more enriched terms with higher statistical significance (Figure 3D), whereas pseudotimeDE does not yield any significant enrichment in GO biological processes. Meanwhile, the enriched GO terms of the MIGC results on this dataset show substantial overlap with those obtained from the first mouse brain dataset (Figure 2F). In addition, an ablation study across subsampling rates of 0.75, 0.5, and 0.25 further demonstrates the robustness of MIGC across subsampling (Supporting Information S1: Figure S2). In conclusion, MIGC can infer pseudotime from the differentiation process and yield robust findings from multiple mouse brain datasets.

**FIGURE 3:**
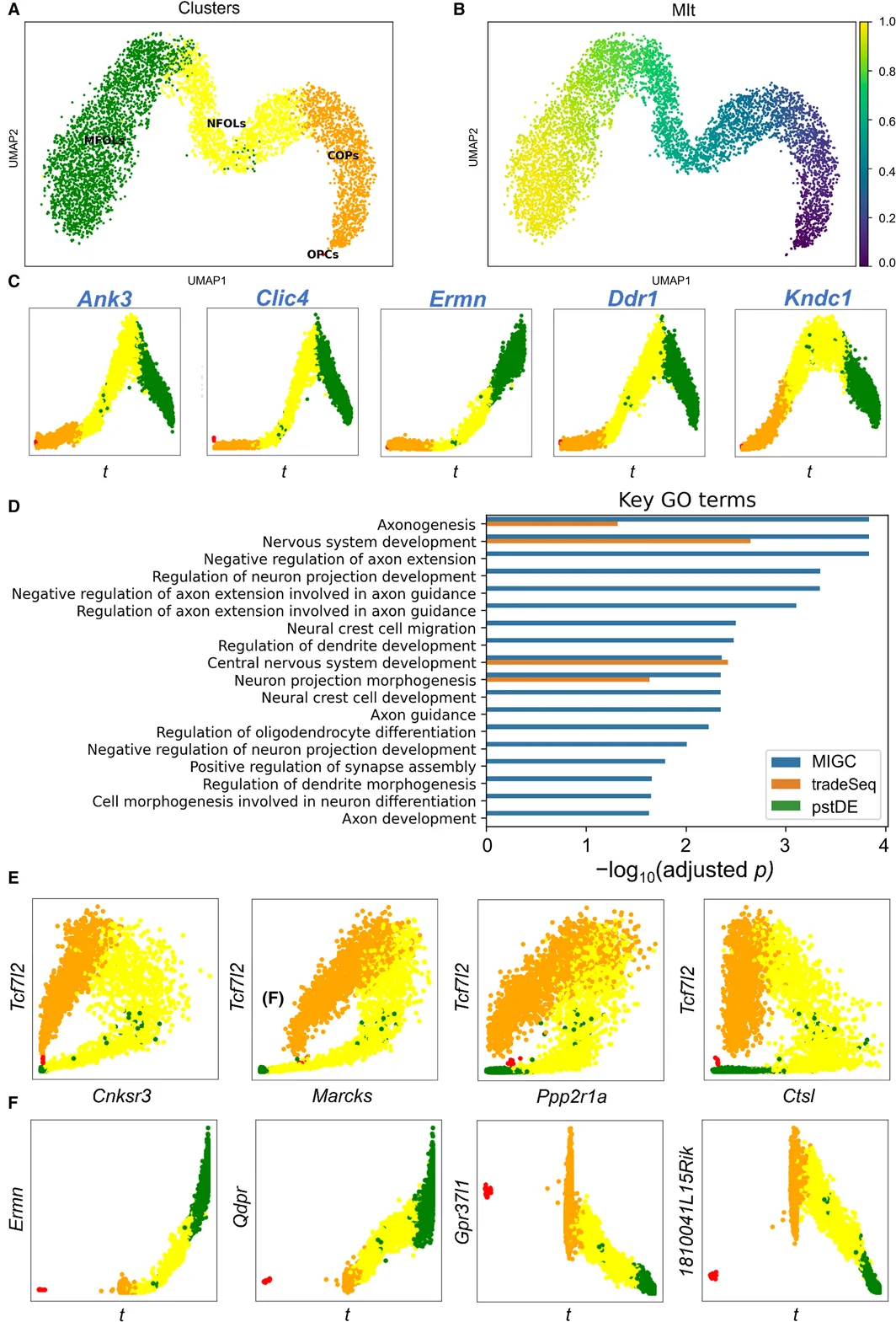
Results of MIGC on the hindbrain of mice. (A) The cell type annotation. (B) The inferred pseudotime representation MIt. (C) The expression of the top‐5‐ranked pseudotime DEGs over time, which are *Ank3*, *Clic4*, *Ermn*, *Ddr1*, and *Kndc1*. (D) The comparison of enriched GO terms based on the top‐100 pseudotime DEGs selected by MIGC, tradeSeq, and pseudotimeDE (pstDE). (E) The coexpression between the transcription factor (TF) *Tcf7l2* and four target genes, including *Cnksr3*, *Marcks*, *Ppp2r1a*, and *Ctsl*. (F) The expression abundance along Palantir pseudotime of the top‐4‐ranked genes selected by MIGC based on the Palantir pseudotime. DEGs, differentially expressed genes.

Furthermore, recent studies on gene regulatory network inference have shown the importance of incorporating temporal patterns into analysis [19, 32, 33, 34]. Utilizing the MIGC method for selecting pseudotime DEGs could facilitate the analysis of time‐delayed regulatory patterns within genes, which could benefit the understanding of gene regulation. Recently, RNA velocity studies [9, 11, 35] have posited that in the presence of causality with a time delay, the joint distribution of two variables should demonstrate a clockwise curve, resembling an almond shape. Inspired by the idea that transcription factors (TFs) and their target genes can also exhibit time‐delayed patterns, we investigated the coexpression patterns between TFs and their targets within the pseudotime DEGs identified by MIGC. As illustrated in Figure 3E, the coexpression pattern between the TF *Tcf7l2* and four genes (*Cnksr3*, *Marcks*, *Ppp2r1a*, and *Ctsl*) exhibits a clear clockwise curve pattern. Notably, all four genes are annotated as target genes regulated by *Tcf7l2* according to the ENCODE TF‐target database [36]. These findings suggest that the expression of these target genes changes following *Tcf7l2* with time delays, supporting regulatory relationships.

Theoretically, the MIGC pseudotime DEG detection approach could be combined with other pseudotime inference methods. To test its performance, we applied the MIGC pseudotime DEG selection strategy to the Palantir [7] pseudotime, identifying the top‐four‐ranked genes: *Ermn*, *Qdpr*, *Gpr37l1*, and *1810041L15Rik*. Palantir pseudotime disturbs the temporal continuity between OPCs and COPs (Figure 3F), whereas the distribution of these genes in the uniform manifold approximation and projection (UMAP) space does not support these patterns. These disruptions in pseudotime could introduce noise into DEG identification. Given that both MIGC pseudotime inference and MIGC gene selection pursue the same objective of maximizing MI, we recommend performing MIGC pseudotime DEG detection based on MIGC pseudotime inference.

### 2.4 MIGC can detect the pseudotime DEG set in the erythroid development process

On a gastrulation erythroid dataset selected from the transcriptional profile of mouse embryos [37], the pseudotime inferred by MIGC fits the developmental trajectory in the erythroid lineage well, which is from blood progenitors to erythroid cells (Figure 4A,B). The detected pseudotime DEGs show clear interpretability. For instance, the top‐1‐ranked key gene suggested by MIGC, *Hbb‐bh1* (Figure 4C), has been reported as a key enzyme involved in heme metabolism [38]. The GO enrichment analysis based on the top‐100 pseudotime DEGs further confirms that MIGC can detect the key genes for the development of erythroid cells (Figure 4D). The most significantly enriched GO terms are “heme biosynthetic process” and “porphyrin‐containing compound biosynthetic process,” whose adjusted *p*‐values are about $10^{-10}$. In vertebrates, heme, a porphyrin‐containing compound, is a key component of hemoproteins, which are the primary components of red blood cells.

**FIGURE 4:**
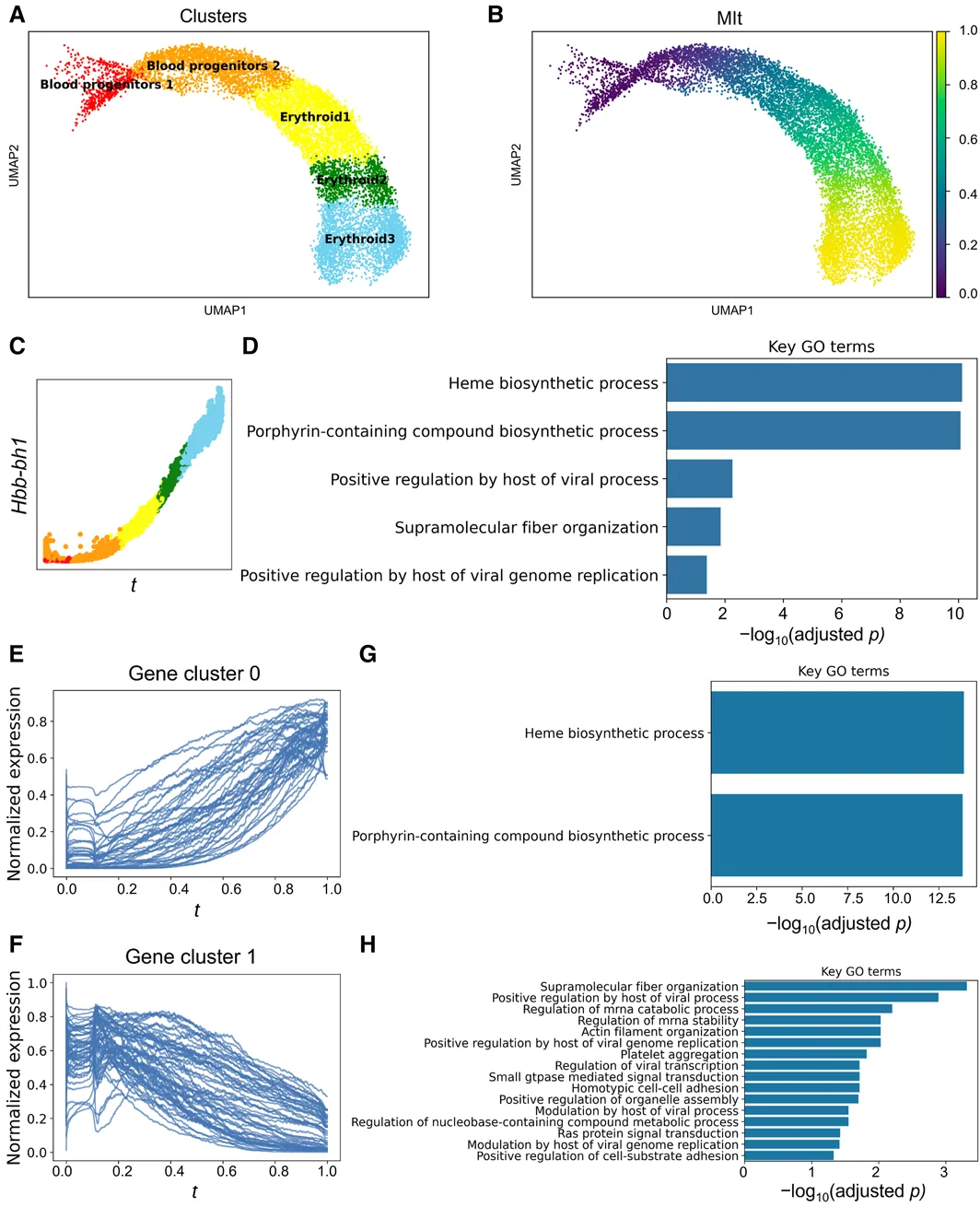
Results of MIGC on the gastrulation erythroid dataset. (A) The cell type annotation. (B) The inferred pseudotime representation MIt. (C) The expression of *Hbb‐bh1* over time, which is the top‐ranked pseudotime‐related key gene. (D) GO term enrichment analysis based on pseudotime differentially expressed genes. (E) The expression patterns along pseudotime of genes in Cluster 0. (F) The expression patterns along pseudotime of genes in Cluster 1. (G) GO term enrichment analysis of genes in Cluster 0. (H) GO term enrichment analysis of genes in Cluster 1.

To further analyze these pseudotime DEGs, we performed clustering on them and found two groups of genes according to their expression patterns along pseudotime. The 42 genes in Cluster 0 are upregulated during differentiation (Figure 4E), whereas the other 58 genes in Cluster 1 are downregulated (Figure 4F). We next performed the GO enrichment analysis for genes in both clusters. For Cluster 0, two GO terms are significantly enriched, which are “heme biosynthetic process” and “porphyrin‐containing compound biosynthetic process” again (Figure 4G). Their adjusted *p*‐values are about $10^{-13}$, even lower than those obtained from all pseudotime DEGs (Figure 4D). On the contrary, although the expression patterns of genes in Cluster 1 are similar to each other, the enriched GO terms are much less significant and are not concentrated on specific functions (Figure 4H). In mature red blood cells, which mainly consist of hemoproteins, the nucleus, ribosomes, and other organelles are degenerated. As a result, during the differentiation of erythroid cells, the upregulated genes (Cluster 0) would significantly enrich in heme‐associated biological processes, and the downregulated genes (Cluster 1) could have various functions without specific enrichment. Details about those 42 genes in Cluster 0, which are likely to be pseudotime DEGs in erythroid development, are provided in Supporting Information S1: Table S1 and Figure S3. An ablation study on the hyperparameters of MIGC is provided in Supporting Information S1: Table S2.

## 3 DISCUSSIONS

Recent advances in single‐cell transcriptomics have enabled detailed characterization of temporal dynamics at cellular resolution, where pseudotime inference and identification of pseudotime DEGs are central tasks. Here, we present MIGC, a trajectory analysis framework that maximizes MI between gene expression and cellular states. MIGC jointly promotes consistent pseudotime inference and robust DEG identification under a unified objective. We validated MIGC across multiple independent datasets (results on two additional datasets are provided in Supporting Information S1: Figure S4), demonstrating improved performance over baseline approaches.

Despite these strengths, MIGC has several limitations. First, the current framework is not specifically designed for multibranch trajectories and requires manual specification of the initial cell cluster. This limitation could be alleviated by leveraging unspliced/spliced counts or developmental potential [39] measures to infer the initial state, followed by branch identification from this root based on the connectivity graph within cell clusters [40]. In addition, iterative or co‐optimization strategies could be explored, where pseudotime inference and gene selection are updated in a feedback loop, potentially further improving internal consistency and robustness in capturing cellular dynamics.

## 4 CONCLUSION

In this study, we developed MIGC, an MI‐based framework for joint pseudotime inference and pseudotime differential gene detection from scRNA‐seq data. By directly modeling the dependence between gene expression and cellular progression, MIGC provides a unified and interpretable approach to trajectory analysis. Benchmarking across multiple datasets showed that MIGC accurately reconstructs pseudotemporal ordering and consistently identifies biologically relevant genes associated with dynamic cellular processes. These results demonstrate that MIGC offers an effective alternative to conventional pseudotime analysis pipelines and provides a useful framework for investigating gene expression dynamics during cell differentiation and development.

## 5 METHODS

### 5.1 Data preprocessing

We performed preprocessing on the scRNA‐seq datasets. First, the counts in each cell were normalized by total counts over all genes, which was followed by log transformation. Then, we selected the 2000 HVGs using Seurat v3 [41]. After performing principal component analysis, a nearest‐neighbor graph (with 30 neighbors by default) was constructed based on Euclidean distances in the top‐30 principal component space among cells. For scRNA‐seq datasets, the moments were computed for each cell across its nearest neighbors, which can smooth the expression and reduce data sparsity.

### 5.2 The estimation of RSMI

MI is widely adopted for measuring the correlation between two random variables, as it can capture nonlinear dependencies from the data. For two random variables X and Y, MI is defined as the Kullback–Leibler (KL) divergence between their joint distribution and the product of their marginal distributions:

(1) $$\begin{array}{c} \text{MI}\left(X,Y\right)=\text{KL}\left(p\left(x,y\right),p\left(x\right)p\left(y\right)\right)=E_{p_{xy}}\left[\log\frac{p\left(x,y\right)}{p\left(x\right)p\left(y\right)}\right] \end{array}.$$

where *E* means the mathematical expectation and *p* means the probability distribution. Although MI is theoretically a powerful measurement in data science, when dealing with continuous variables whose probability distributions are unknown, exact computation of MI is not feasible [42]. In this study, an extraordinarily fast estimation of MI for one‐dimensional variables is required because we need to calculate MI for each sample in each iteration. Therefore, we introduce RSMI and its estimation ERSMI [20], which can meet our requirements.

Like MI, RSMI can also measure how two random variables are related. RSMI is defined as the relative Pearson divergence (RPE) between the joint distribution and the product of their marginal distributions:

(2) $$\begin{array}{c} \text{RSMI}\left(X,Y\right)=\text{RPE}\left(p\left(x,y\right),p\left(x\right)p\left(y\right)\right)=E_{q_{xy}}\left[{\left(\frac{p\left(x\right)p\left(y\right)}{q\left(x,y\right)}-1\right)}^{2}\right] \end{array}.$$

where the joint distribution *q* is defined as q(x,y)=βp(x)p(y)+(1−β)p(x,y). β is a hyperparameter, which is set to 0.5 by default in our implementation.

The advantage of RSMI is that it can be estimated with the least squares method, which means that the global optimum can be obtained as an analytical solution. This enables fast and accurate implementation based on tensor computation. For using the least squares method, we first defined the density ratio ω(x,y) as $\omega \left(x,y\right)=\frac{p\left(x\right)p\left(y\right)}{q\left(x,y\right)}$. Then, the density ratio is modeled with a kernel function:

(3) $$\begin{array}{c} \omega_{\alpha }\left(x,y\right)=\alpha^{T}\varphi \left(x,y\right)=\alpha^{T}\left(k\left(x\right)\;\circ \;l\left(y\right)\right) \end{array}.$$

where ∘ denotes the Hadamard product. For calculation efficiency, the kernel function $\varphi \left(x,y\right)\in R^{m}$ is factorized as the product of $k\left(x;x_{c}\right)\in R^{m}$ and $l\left(y;\;y_{c}\right)\in R^{m}$, which are kernel functions of *x* and *y*, respectively. $x_{c}\in R^{m}$ and $y_{c}\in R^{m}$ are the centers of the kernel functions k and l, respectively. Parameters $\alpha \in R^{m}$ and m are the number of kernels. The squared error of the density ratio is defined as follows:

(4) $$\begin{array}{c} J\left(\alpha \right)=E_{q_{xy}}\left[{\left(\omega_{\alpha }\left(x,y\right)-\omega \left(x,y\right)\right)}^{2}\right] \end{array}.$$

Approximating the expectations of H and h by empirical averages, the objective function becomes as follows:

(5) $$\begin{array}{c} \hat{J}\left(\alpha \right)=\left[\alpha^{T}\hat{H}\alpha -2{\hat{h}}^{T}\alpha +\lambda \alpha^{T}I\alpha \right] \end{array},$$

where a regularization term $\lambda \alpha^{T}I\alpha$ is included to avoid overfitting with λ= 0.001, $I\in R^{m\times m}$ is an identity matrix, and

(6) $$\begin{array}{c} \hat{H}=\frac{1\;-\;\beta }{n}\left(K\;\circ \;L\right){\left(K\;\circ \;L\right)}^{T}+\frac{\beta }{n^{2}}\;\left(KK^{T}\right)\circ \left(LL^{T}\right) \end{array}.$$

(7) $$\begin{array}{c} \hat{h}=\frac{1}{n^{2}}\left(K1_{n}\right)\;\circ \;\left(L1_{n}\right), \end{array}$$

where $K=\left[k\left(x_{1};x_{c}\right)\cdot \cdot \cdot k\left(x_{n};x_{c}\right)\right],L=\left[l\left(y_{1};y_{c}\right)\cdot \cdot \cdot l\left(y_{n};y_{c}\right)\right]$, n is the number of samples, and 1*n* means an *n*‐dimensional vector filled with ones. Differentiating the above objective function with respect to α and equating it to zero, we can obtain the optimal solution for $\hat{\alpha }$ analytically as follows:

(8) $$\begin{array}{c} \hat{\alpha }={\left(\hat{H}+\lambda I\right)}^{-1}\hat{h} \end{array}.$$

Finally, ERSMI can be obtained by the following equation:

(9) $$\begin{array}{c} \text{ERSMI}=2{\hat{\alpha }}^{T}\hat{h}-{\hat{\alpha }}^{T}\hat{H}\hat{\alpha }-1 \end{array}.$$

In our implementation, Gaussian kernels are employed in k(x) and l(y). ERSMI could be directly obtained as the analytical solution through matrix computation, which can provide gradients for implementation in neural network models with PyTorch and also support GPU acceleration for further improving time efficiency. Specifically, continuous variables are projected onto a set of uniformly spaced kernel centers defined over $\left[\mu_{\min},\mu_{\max}\right]$ with step size $\mu_{\text{step}}$, and Gaussian kernels with bandwidth σ are used to construct feature mappings. In practice, $\mu_{\min}=0$, $\mu_{\max}=1$, and $\mu_{\text{step}}=0.1$ are used, resulting in a fixed grid resolution that balances estimation accuracy and computational cost. The kernel bandwidth σ controls the smoothness of the density approximation and is set to 0.05 by default. To ensure numerical stability in matrix inversion, a small diagonal regularization term ($10^{-3}$) is added. All computations are performed in a batched manner using GPU acceleration to improve efficiency. The pseudocode of the ERSMI calculation is provided in the supplementary information.

We computed MI between gene expression and pseudotime using both ERSMI and the function provided in the scikit‐learn package of Python, which was conducted for each of the 2000 HVGs from the pancreas scRNA‐seq data. As shown in Supporting Information S1: Figure S1, ERSMI is highly consistent with MI (Spearman’s *ρ* > 0.99; Supporting Information S1: Figure S1A) and is more than 200 times faster than MI (Supporting Information S1: Figure S1B). Consequently, ERSMI, which could be regarded as a fast estimator of MI, is utilized in this study when MI between two continuous variables is required.

### 5.3 The generation of pseudotime

Using the preprocessed expression data, we first performed cell clustering with the Leiden algorithm, followed by the computation of MI between each gene and the Leiden clusters. Subsequently, we selected the top‐200 genes with the highest MI scores, which were input into the neural network. Furthermore, consistent with numerous pseudotime inference methods [4, 5, 6, 7], MIGC also requires manual annotation of the initial cluster for generating the pseudotime representation MIt.

The generation of MIt is facilitated through a simple two‐layer neural network, which comprises two fully connected layers with a latent size of 128 and the exponential linear unit activation function. The output is a one‐dimensional embedding that is constrained within the range of 0–1 using the sigmoid function. The model undergoes 500 epochs of training, utilizing the Adam [43] optimizer with a learning rate set to 0.001. The loss function is the negative mean MI between the input 200 genes and the output cell embedding, where the MI is estimated using ERSMI. These model specifications and hyperparameters are consistent across all scRNA‐seq datasets. After the training procedure, the optimized cell representation MIt could be obtained, which has the maximized MI with the input gene expressions. Then, we input the expression levels of the 200 selected genes into the model once more to generate cell state representations, with the output subsequently rescaled to the range of 0–1. Utilizing the annotated initial cluster, we can determine whether the model learned a completely inverse pseudotime, which needs to be corrected by reversing the representation. Finally, the pseudotime representation MIt is learned by MIGC.

### 5.4 The selection of pseudotime DEGs

After acquiring the pseudotime MIt, we can calculate ERSMI between each gene’s expression and the learned cell representation for all 2000 HVGs. Subsequently, the top‐100 genes with the highest ERSMI scores are defined as the pseudotime DEGs, which could be utilized for downstream enrichment analysis of GO terms.

### 5.5 GO term enrichment analysis

The enrichment analysis of GO terms for biological processes is performed utilizing the gseapy.enrichr function within the Python package gseapy. This function defaults to employing Fisher’s exact test. In each dataset, the background gene set is defined as the selected 2000 HVGs after preprocessing.

### 5.6 Baseline implementation

tradeSeq: According to the tutorial provided on the tradeSeq website, we first perform Slingshot for pseudotime inference and then use the “fitGAM” function to fit gene expression as a function of time. After that, the “associationTest” function, which is based on the Wald test, is utilized for pseudotime DEG detection. The pseudotime DEGs are sorted according to the Wald test statistics. We then selected the top‐100 genes with the highest Wald test statistics for downstream enrichment analysis of GO terms.

pseudotimeDE: We followed the standard pseudotimeDE pipeline, including normalization, selection of HVGs, PCA‐based dimensionality reduction, and pseudotime inference using Slingshot [6]. pseudotimeDE was then applied to the 2000 HVGs to assess their association with pseudotime, and genes were ranked according to the adjusted *p*‐values. We then selected the top‐100 genes for downstream GO enrichment analysis.

## AUTHOR CONTRIBUTIONS

**Jiachen Li:** Conceptualization; methodology; software; writing; visualization. **Ye Yuan**: Supervision; writing—review and editing.

## CONFLICT OF INTEREST STATEMENT

The authors declare no conflicts of interest.

## ETHICS STATEMENT

This study is a computational analysis based on publicly available datasets and did not involve human participants or animal subjects. Therefore, no additional ethical approval was required.

## Supporting information

Supporting Information S1

## Data Availability

The 10× embryonic mouse brain dataset used in this study is provided on the GitHub website (github.com/lijc0804/MIGC/blob/main/data/10x_mouse_brain/adata_rna.h5ad). The raw data of the hindbrain (pons) of adolescent mice are from the website (pklab.med.harvard.edu/ruslan/velocity/oligos/). The gastrulation erythroid dataset, which is selected from the transcriptional profiles of mouse embryos [37], is incorporated by scvelo.datasets.gastrulation_erythroid(). Baseline approaches’ tutorials for tradeSeq and pseudotimeDE are available on the GitHub websites (statomics.github.io/tradeSeq/articles/tradeSeq.html and github.com/SONGDONGYUAN1994/PseudotimeDE). The source code of this study can be downloaded from the GitHub repository (github.com/lijc0804/MIGC).
