# Supplementary material for: MIGC: Pseudotime differentially expressed genes detection with mutual information
Source: Quant Biol. 2026 Oct 19;14(4):e70057. doi: 10.1002/qub2.70057 (PMC13681047; doi:10.1002/qub2.70057)
Supplement: Supplementary file 1 [file QUB2-14-e70057-s001.docx]

**Supplementary Information of MIGC**

**Relations between entropy and correlation**

**Figure S1. The comparison between entropy and variance of the genes’ expression on four scRNA-seq datasets.** (A) 10x mouse brain. (B) hindbrain (pons) of adolescent mice. (C) gastrulation erythroid. (D) pancreas.

**Pseudocode of the ERSMI computation**

Algorithm: Batch ERSMI Estimation

Input:

I1, I2	# two continuous variables (batch × features)

σ	# Gaussian kernel bandwidth

μ_min, μ_max	# kernel center range

μ_step	# step size for kernel centers

ε	# small regularization term

Output:

ERSMI score per batch

1. Construct kernel centers:

μ ← {μ_min, μ_min + μ_step, ..., μ_max}

n ← number of centers

2. Form 2D grid of centers:

(μ_x, μ_y) ← all pairwise combinations of μ

total centers = n × n

3. Compute kernel feature mappings:

For each sample in batch:

K ← exp(−(I1 − μ_x)² / (2σ²))

L ← exp(−(I2 − μ_y)² / (2σ²))

4. Compute matrices:

Compute H and h according to equation (6) and (7) in the manuscript

5. Stabilize matrix:

H ← H + ε I

6. Solve for coefficients:

α ← H⁻¹ h

7. Compute ERSMI:

ERSMI ← 2 αᵀ h − αᵀ H α − 1

**The validation of consistency across subsampling for MIGC**

On the mouse pons dataset, we evaluated the robustness of MIGC under subsampling by randomly selecting subsets of cells during preprocessing. The subsampling ratios were set to 0.75, 0.5, and 0.25. For each subset, we applied MIGC to perform pseudotime inference, identify DEGs, and conduct GO enrichment analysis. The comparison of key GO terms (defined as those related to the nervous system) enriched from the top 100 ranked genes is shown in Figure S2. The results indicate that MIGC is highly consistent across different subsampling levels, with only minor variations observed.

**Figure S2. The validation of consistency across subsampling for MIGC on the mouse pons dataset. The subsampling rates are 0.75, 0.5 and 0.25, respectively.**

**Additional results on the erythroid dataset**

**Table S1. The 42 pseudotime DEGs which are up-regulated in the erythroid development process.**

| **Gene** | **ERSMI** | **Gene** | **ERSMI** | **Gene** | **ERSMI** |
| --- | --- | --- | --- | --- | --- |
| *Hbb-bh1* | 0.666 | *Mllt3* | 0.553 | *Hebp1* | 0.493 |
| *Blvrb* | 0.646 | *Trim10* | 0.548 | *Rfesd* | 0.489 |
| *Gypa* | 0.641 | *Alas2* | 0.546 | *Slc25a37* | 0.487 |
| *Tmem14c* | 0.638 | *Mrap* | 0.545 | *Cpox* | 0.483 |
| *Alad* | 0.619 | *Fech* | 0.537 | *Ppox* | 0.476 |
| *Hmbs* | 0.61 | *Smim1* | 0.532 | *Gmpr* | 0.473 |
| *Gpx1* | 0.605 | *Atpif1* | 0.525 | *Uros* | 0.473 |
| *Acp5* | 0.599 | *Chchd10* | 0.51 | *Svbp* | 0.47 |
| *Hba-x* | 0.596 | *Glrx5* | 0.507 | *Fth1* | 0.461 |
| *Prdx2* | 0.595 | *Hba-a2* | 0.505 | *Klf1* | 0.46 |
| *Pnpo* | 0.582 | *Car2* | 0.497 | *Hemgn* | 0.459 |
| *Mgst3* | 0.567 | *Dhrs11* | 0.495 | *Gclm* | 0.458 |
| *Hba-a1* | 0.564 | *Hbq1b* | 0.494 | *Smox* | 0.448 |
| *Urod* | 0.557 | *Fam210b* | 0.493 | *Rragd* | 0.448 |

**Figure S3. The 42 pseudotime DEGs which are up-regulated in the erythroid development process.**

**The application of MIGC on more datasets**

On a dataset of E15.5 mouse pancreas^4,5^, the inferred pseudotime MIt can identify the differentiation process, which starts from ductal cells and terminates at endocrine cells, including alpha, beta, gamma, and epsilon cells (Figure S4A). The top-5 key genes selected by MIGC are *Ypel3*, *Map1b*, *Rpl18a*, *Rps8* and *Npm1*, all of which show clear dynamics along pseudotime (Figure S4B).

On a scRNA-seq dataset of human preimplantation embryos ranging from embryonic (HESC) day 3 to 7^6^, the inferred pseudotime can detect the correct time order between cells collected from different time points (Figure S4C). The top-ranked key genes selected by MIGC include *Khdc1l*, *Hint1*, *Dlx3*, *Nlrp7* and *Ptn*, all of which also show clear dynamics along pseudotime (Figure S4D). Based on the results of all these datasets, we can conclude that MIGC provides a robust approach for pseudotime inference and pseudotime-related key genes detection.

**Figure S4. The Results on various datasets.** (A) Cell annotation and pseudotime inferred by MIGC on a pancreas dataset. (B) The expression of *Ypel3*, *Map1b*, *Rpl18a*, *Rps8* and *Npm1* along the MIGC pseudotime on pancreas dataset, which are the top-5 ranked key genes selected by MIGC. (C) Cell annotation and pseudotime inferred by MIGC on the HESC scRNA-seq dataset. (D) The expression of *Khdc1l*, *Hint1*, *Dlx3*, *Nlrp7* and *Ptn* along the MIGC pseudotime on the HESC dataset, which are the top-ranked key genes selected by MIGC.

The pancreatic endocrinogenesis dataset^5^ comprises the single-cell RNA-seq (10×) data sampled from mouse embryonic day 15.5, which could be acquired from scvelo.datasets.pancreas().

Human preimplantation embryos dataset^6^ is a single-cell RNA-seq dataset can be downloaded with the accession number of E-MTAB-3929 from EMBL-EBI [ebi.ac.uk/biostudies/arrayexpress/studies/E-MTAB-3929].

**The Ablation studies for MIGC**

We conducted a series of ablation studies on the gastrulation erythroid dataset, focusing on (i) the initial gene filtering strategy (number of highly variable genes, n_HVGs), (ii) the number of selected DEGs, and (iii) the choice of model architecture (linear vs. neural network).

Results of this ablation study are shown in **Table S2**, which demonstrate that the biological signal captured by our method is highly robust across different configurations. Under all circumstances, the key biological processes, heme biosynthetic process and porphyrin-containing compound biosynthetic process, remain consistently and highly significantly enriched across all settings tested (*p*-values ranging from 10⁻¹³ to 10⁻¹⁵). Importantly, varying the number of HVGs from 2,000 to 6,000 has negligible impact on enrichment significance, indicating that the downstream results are not sensitive to the initial gene filtering strategy. Similarly, increasing the number of selected DEGs from 100 to 200 leads to only minor fluctuations in *p*-values without altering the overall biological conclusions. This suggests that the method is stable with respect to gene set size selection. Finally, comparing the 4-layer neural network–based formulation with a linear alternative shows that both models produce highly consistent enrichment results across all configurations. In most cases, the *p*-values are nearly identical or differ only slightly, indicating that the neural network component does not introduce instability and that the framework is not overly sensitive to model specification.

Taken together, these ablation results demonstrate that the proposed framework is robust to hyperparameters, including gene preselection, gene set size, and model architecture. Rather than being dependent on any single component, the method consistently captures biologically meaningful signals across a range of reasonable parameter settings, thereby supporting its methodological reliability and practical applicability.

**Table S2**. The ablation study on gastrulation erythroid dataset.

| **GO Term** | **Model type** | ***n*_HVGs** | ***n*_DEGs** | ***p*-value** |
| --- | --- | --- | --- | --- |
| Heme biosynthetic process | Linear | 2,000 | 100 | 8.86E-14 |
| Heme biosynthetic process | NN (4-layer) | 2,000 | 100 | 8.86E-14 |
| Heme biosynthetic process | Linear | 2,000 | 200 | 4.64E-15 |
| Heme biosynthetic process | NN (4-layer) | 2,000 | 200 | 3.71E-13 |
| Heme biosynthetic process | Linear | 4,000 | 100 | 8.86E-14 |
| Heme biosynthetic process | NN (4-layer) | 4,000 | 100 | 8.86E-14 |
| Heme biosynthetic process | Linear | 4,000 | 200 | 4.64E-15 |
| Heme biosynthetic process | NN (4-layer) | 4,000 | 200 | 3.71E-13 |
| Heme biosynthetic process | Linear | 6,000 | 100 | 8.86E-14 |
| Heme biosynthetic process | NN (4-layer) | 6,000 | 100 | 8.86E-14 |
| Heme biosynthetic process | Linear | 6,000 | 200 | 4.64E-15 |
| Heme biosynthetic process | NN (4-layer) | 6,000 | 200 | 3.71E-13 |
| Porphyrin-containing compound biosynthetic process | Linear | 2,000 | 100 | 2.02E-13 |
| Porphyrin-containing compound biosynthetic process | NN (4-layer) | 2,000 | 100 | 2.02E-13 |
| Porphyrin-containing compound biosynthetic process | Linear | 2,000 | 200 | 1.38E-14 |
| Porphyrin-containing compound biosynthetic process | NN (4-layer) | 2,000 | 200 | 9.58E-13 |
| Porphyrin-containing compound biosynthetic process | Linear | 4,000 | 100 | 2.02E-13 |
| porphyrin-containing compound biosynthetic process | NN (4-layer) | 4,000 | 100 | 2.02E-13 |
| porphyrin-containing compound biosynthetic process | Linear | 4,000 | 200 | 1.38E-14 |
| Porphyrin-containing compound biosynthetic process | NN (4-layer) | 4,000 | 200 | 9.58E-13 |
| Porphyrin-containing compound biosynthetic process | Linear | 6,000 | 100 | 2.02E-13 |
| Porphyrin-containing compound biosynthetic process | NN (4-layer) | 6,000 | 100 | 2.02E-13 |
| Porphyrin-containing compound biosynthetic process | Linear | 6,000 | 200 | 1.38E-14 |
| Porphyrin-containing compound biosynthetic process | NN (4-layer) | 6,000 | 200 | 9.58E-13 |

**References**

1	Paninski, L. Estimation of entropy and mutual information. *Neural computation* **15**, 1191-1253 (2003).

2	Belghazi, M. I. *et al.* in *International conference on machine learning.* 531-540 (PMLR).

3	Guo, J. *et al.* in *Proceedings of the IEEE/CVF Conference on Computer Vision and Pattern Recognition.* 18249-18259.

4	La Manno, G. *et al.* RNA velocity of single cells. *Nature* **560**, 494-498 (2018).

5	Bastidas-Ponce, A. *et al.* Comprehensive single cell mRNA profiling reveals a detailed roadmap for pancreatic endocrinogenesis. *Development* **146**, dev173849 (2019).

6	Chu, L.-F. *et al.* Single-cell RNA-seq reveals novel regulators of human embryonic stem cell differentiation to definitive endoderm. *Genome biology* **17**, 1-20 (2016).
